# Acute vertigo: stroke or not?

**DOI:** 10.1097/WCO.0000000000001444

**Published:** 2025-11-24

**Authors:** Diego Kaski

**Affiliations:** SENSE Research Unit, Department of Clinical and Movement Neurosciences, UCL Institute of Neurology, London WC1N 3BG, UK

**Keywords:** acute vertigo, acute vestibular syndrome, dizziness, gait ataxia, labyrinthine stroke

## Abstract

**Purpose of review:**

Acute vertigo accounts for about 4% of emergency department visits in both the United States and Europe. Despite this frequency, the management of dizziness, vertigo, and balance disorders remains fragmented, with no established international care pathway. The acute vestibular syndrome (AVS) is particularly challenging, and timely recognition is essential to avoid potentially devastating outcomes. This review is timely, because misdiagnosis rates remain unacceptably high, especially for posterior circulation strokes presenting with dizziness.

**Recent findings:**

The literature highlights a wide differential diagnosis for AVS, ranging from benign peripheral vestibular disorders to life-threatening central causes. Distinguishing stroke from peripheral disorder remains a key clinical dilemma, compounded by the limitations of early neuroimaging – MRI can yield false negatives within 48 h. Up to 35% of posterior circulation strokes with dizziness are initially missed, often by nonspecialists unfamiliar with targeted bedside tests.

**Summary:**

A structured bedside approach, focusing on key clinical features and targeted examination, can improve diagnostic accuracy and reduce delays in appropriate treatment. Incorporating such strategies into standard practice could address a major gap in acute neurology care and improve patient outcomes.

## INTRODUCTION

Acute dizziness in the emergency department (ED) accounts for approximately 4% of visits in the United States [1] and Europe [2]. The management of dizziness, vertigo and balance disorders is complicated by a lack of a clear internationally accepted pathway. The possible causes of ‘dizziness’ are myriad so many nonspecialist clinicians struggle to decide whether to refer a patient with dizziness to Ear, Nose, and Throat (ENT) surgeons, AudioVestibular Medicine (AVM) physicians, audiology, physiotherapy, neurology, cardiology, gerontology, falls clinics or stroke and emergency departments for acute presentations. In vestibular neurology, there are broadly three clinical presentations: the patient with a single acute vertigo episode, the patient with recurrent dizziness or vertigo and the patient with chronic feelings of dizziness or unsteadiness. In the context of a single acute episode, the pressing question is: does this patient have a primary vestibular disorder such as vestibular neuritis, benign paroxysmal positional vertigo (BPPV) or vestibular migraine, or could this patient have a potentially sinister neurological disorder such as a brain tumour, multiple sclerosis or a posterior circulation stroke? This latter presentation is perhaps the most challenging, partly because of the need for quick action, the lack of trained specialists to assist with emergency decisions and the evolving nature of the symptoms.

The acute vestibular syndrome (AVS) is defined as sudden onset, continuous vertigo lasting for more than 24 h with associated nystagmus, nausea and vomiting, all of which are worsened with head movement [3]. Differentiating between peripheral and central causes can be challenging in the acute phase. In addition, recent studies highlight the presence of false-negative ‘gold standard’ MRI scans in posterior circulation strokes in the first 48 h following symptom onset [4]. Overall, roughly 9% of cerebrovascular events are missed at the initial emergency department presentation and risk of misdiagnosis is much greater when the presenting neurologic complaints are mild, nonspecific, or transient (range 24–60%) [5]. For posterior circulation strokes presenting with dizziness, frontline misdiagnosis appears common, occurring in roughly 35% of cases [6^▪^].

Here, we will discuss the practical approach to a patient with acute vertigo and focus on the features that would facilitate the diagnosis of stroke. 

**Box 1:**
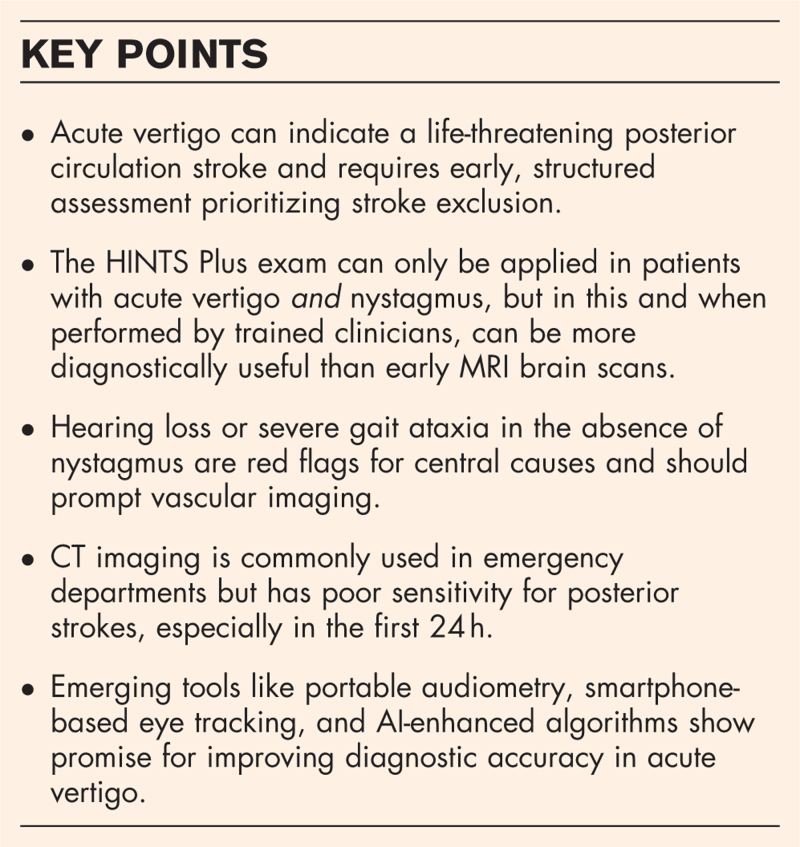
no caption available

## THE CLINICAL HISTORY

A brief clinical history is a good starting point. Note that this differs from the need for a detailed clinical history in a patient with chronic dizziness. First, one needs to try to understand whether the patient is describing vertigo (that localizes to the vestibular network) or nonvertiginous dizziness (e.g. headache, light-headedness), or perhaps even a gait disorder (e.g. early parkinsonism or cerebellar disease). To do this, ask the patient to describe their symptoms without the use of the word ‘dizziness’. If they struggle, you may need to ask more probing questions such as ‘is the room spinning, do you feel you are spinning, is it a sense of faintness, or unsteadiness?’ Next, think about possible triggers to their dizziness (e.g. turning over in bed, head injury, life stressors and starting a new medication). Then, enquire about associated features, such as light or sound sensitivity, motion sensitivity (all of which point to migraine), or other neurological symptoms, such as diplopia, dysarthria, dysphagia or extreme unsteadiness that suggests a neurological (rather than inner ear) disorder. Indeed, it is useful to directly seek sigs of stroke at an early stage rather than thinking about these at the end of the assessment. It is useful to explore vascular risk factors, as these will raise the probability of stroke. Finally, ask about hearing. Loss of hearing in one ear is a red flag from stroke, but neurologists are not always good at exploring this, probably because it has traditionally been considered an ENT symptom [7].

## THE CLINICAL EXAMINATION

**FIGURE 1:**
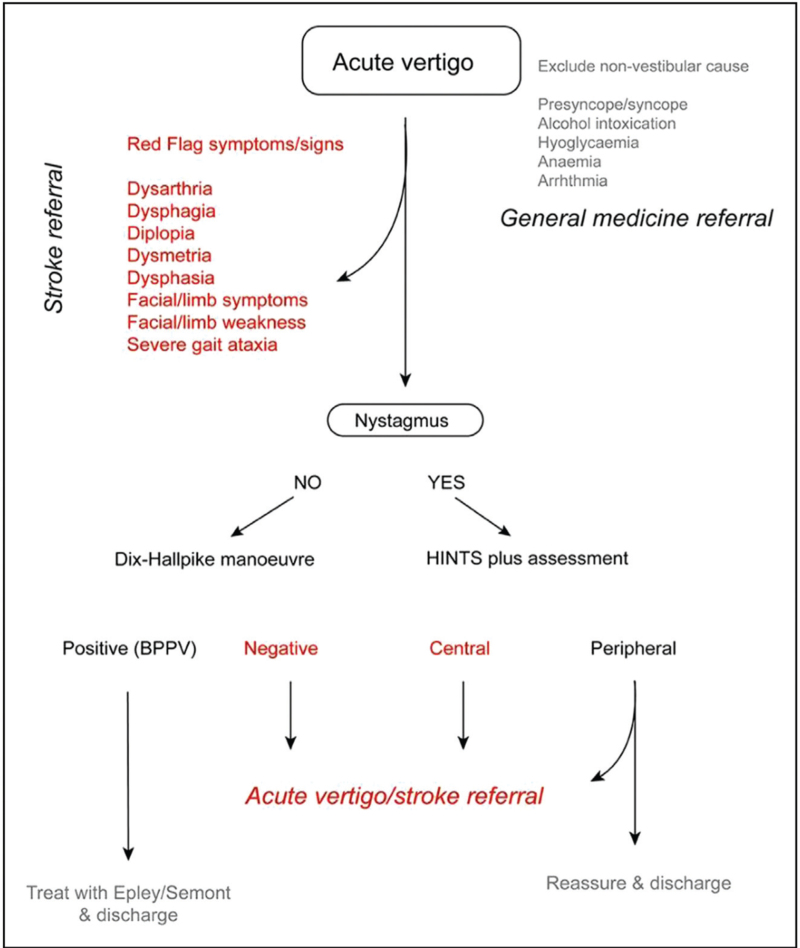
Diagnostic algorithm for acute vertigo, emphasizing the need to ‘rule in’ stroke first, rather than focusing only on the eye movement examination. Data from [24].

As for the clinical history, it is good practise to start by looking for signs of stroke, dysarthria, dysphasia, ataxia, facial and/or limb weakness. Figure 1 provides a proposed algorithm for the assessment of a patient with acute vertigo. Note that actively identifying signs of stroke or other obvious causes of vertigo (e.g. anaemia, hypoglycaemia and orthostatic hypotension) should come first. You can then move onto more specific signs to identify stroke if other signs are not present.

The bedside vestibular evaluation in a patient with acute vertigo focuses on eye movements, hearing and gait assessment. The components of the eye movement examination are ocular alignment, range of movement, fixation and gaze holding, smooth pursuit and saccades, vestibular ocular reflex (VOR) and VOR suppression. It is also important to perform a Dix–Hallpike manoeuvre, to look for BPPV and central positional nystagmus, a feature of some posterior circulation stroke syndromes.

In patients with spontaneous nystagmus, it is useful to apply the HINTS bedside assessment. The HINTS exam (Head Impulse, Nystagmus, Test of Skew) is a bedside neurological examination used to differentiate between peripheral and central causes of acute vertigo [5]. HINTS plus adds an assessment of auditory function (hearing) to the HINTS exam that increases the sensitivity in picking up stroke involving arterial supply to the labyrinth. Note the HINTS plus will either suggest a peripheral vestibular syndrome (e.g. vestibular neuritis) or a central vestibular syndrome (e.g. stroke), but is always ‘abnormal’ because there will be nystagmus; to recap, in the absence of nystagmus, the HINTS plus cannot be applied. Neurologists tend not to examine hearing formally (how many of us have said ‘cranial nerves II-XII intact’ without actually probing hearing!).

Severe difficulty standing is a key feature of central vestibular disorder, often under-recognized in AVS present in 55% of patients with AVS of central cause [8], although in most studies of AVS, the focus has been on eye movements, and truncal ataxia has not been formally evaluated. In one study, 15% of patients with acute truncal ataxia (ATA) lacked nystagmus but had central causes such as cerebellar or thalamic stroke, multiple sclerosis or tumour. Infarcts in specific territories (e.g. posterior inferior cerebellar artery – PICA, anterior inferior cerebellar artery – AICA, superior cerebellar artery – SCA, and thalamus) can cause gait ataxia without nystagmus [8]. In such scenarios, smooth pursuit and saccadic abnormalities were found in some older patients, but their diagnostic utility was limited. The key message is that gait assessment is crucial, especially when nystagmus is absent.

### Pitfalls of diagnostic eye movement examination

Examination of eye movements at the bedside – including the Dix–Hallpike test and the HINTS-plus protocol – depends on a level of patient cooperation that may be difficult to achieve during episodes of hyperacute vertigo. Clinicians without specialist expertise often require more time to detect subtle ocular motor abnormalities [9]. Nonetheless, using structured assessment protocols can enhance diagnostic accuracy; for example, the Dix–Hallpike remains a cornerstone in confirming BPPV [10] but also useful for picking up central (positional) nystagmus that could support a stroke diagnosis. Common challenges during eye movement evaluation can be partially addressed by optimizing the clinical environment – such as minimizing external distractions, ensuring sufficient lighting and modifying manoeuvres to suit spatial constraints (e.g. using the side-lying Dix–Hallpike technique) [11]. Consistent use of these standardized methods, supported by targeted training, may lead to better diagnostic performance. Currently, emergency department practitioners typically lack formal instruction in ocular motor assessment, though many acknowledge the potential benefits such training could provide [12].

## ACUTE VESTIBULAR SYNDROME: PERIPHERAL UNILATERAL PERIPHERAL VESTIBULOPATHY (VESTIBULAR NEURITIS)

Vestibular neuritis is one of the three most common causes of acute vertigo. It can be thought of as an acute unilateral vestibular ‘paresis’ and has variously been known as vestibular neuronitis, vestibular neuro-labyrinthitis or acute peripheral vestibulopathy [13]. The syndrome is also often referred to as labyrinthitis, considered a misnomer when there is neither hearing loss nor tinnitus, because the condition nearly always spares the cochlear nerve. Our preference in this scenario is to use the term acute peripheral vestibular loss, when the exact diagnosis is not known, it is only presumed to be related to an inflammatory process of the vestibular nerve. Importantly, additional symptoms of hearing loss or tinnitus if present with acute vertigo are red flags for a vascular event, so it seems prudent to actively search a vascular event in such patients to prevent significant stroke events in the future. This is the reason that the introduction of HINTS plus has shown better diagnostic accuracy where the ‘plus’ accounts for sudden onset asymmetric hearing loss [14].

Vestibular neuritis typically presents with a sudden onset of spinning vertigo, oscillopsia, imbalance, nausea or vomiting, and no accompanying auditory or broader neurological symptoms. Diagnosis can be confidently established when key ocular motor findings are present: namely, a unidirectional horizontal nystagmus (often with a minor torsional component) beating away from the affected ear, a positive head impulse test towards the lesioned side, and gait unsteadiness. This gait disturbance tends to be moderate – patients are generally able to stand unaided – unlike the pronounced truncal instability seen in some acute cerebellar disorders, such as stroke [15^▪^]. Importantly, the presence of both unidirectional nystagmus and a corresponding abnormal head impulse test constitutes the minimal criteria for diagnosing vestibular neuritis. Any other eye movement abnormalities – including bidirectional (‘gaze-evoked’) horizontal, vertical, or purely torsional nystagmus – or deficits in smooth pursuit, saccadic control, or ocular alignment (e.g. abducens palsy, internuclear ophthalmoplegia or vertical skew) should prompt consideration of a central cause.

The treatment of unilateral (and bilateral) vestibular dysfunction is intensive vestibular rehabilitation physiotherapy [16]. Many patients make a rapid recovery over days without the need for rehabilitation. In patients with high levels of visual dependency (those that tend to keep their eyes on the floor when walking, avoid moving their heads even when the acute vertigo has settled, etc.) and those with high levels of anxiety or body vigilance may need earlier rehabilitation [17]. A range of vestibular rehabilitation programmes has been devised to promote sensitization and gaze stabilization. Every effort should be made to ensure optimal sensory input for balance that may include correction of visual disorders such as cataracts and management of other neurological or orthopaedic problems (e.g. cervical spondylosis, headaches and sleep disturbance). There has been ongoing debate about the role of steroids in such settings, and there is limited evidence to support a meaningful benefit and in the UK, these are not routinely used [18].

## ACUTE VESTIBULAR SYNDROME: CENTRAL

### Stroke or Transient Ischaemic Attack (TIA)

An AVS is a medical emergency, and assessment of the eye movements and gait are the central components of the examination in this context. Patients can be seriously ill with vertigo, unsteadiness, nausea and vomiting to the point of dehydration requiring hospitalization or be at risk of ‘malignant’ transformation of a small brainstem infarct to one that involves the cerebellum also (and could require neurosurgical decompression).

Neuroanatomically, there are three main vascular vertigo syndromes, with ischemic stroke outnumbering haemorrhagic stroke. Those that affect the SCA, PICA, or AICA. SCA syndromes are more typically ‘cerebellar’ (rather than vestibular), with marked limb incoordination, gait ataxia and dysarthria. PICA territory strokes are a more common causes of AVS than AICA, and most affect the cerebellum followed by the medulla, pons and thalamus. As PICA supplies the posterior inferior cerebellum (including the nodulus, uvula and flocculus) and ipsilateral pontomedullary vestibular nucleus, the main symptoms and signs of a PICA territory infarction may consist only of vertigo and truncal ataxia without the other classic cerebellar signs of gaze-evoked nystagmus or dysmetria [6^▪^,^9^]. The presence of acute unilateral hearing loss with AVS suggests that a lateral pontine stroke in the distribution of AICA is somewhat more probable. Head or neck pain with sudden-onset vertigo should prompt evaluation for vertebral dissection.

## IMAGING IN ACUTE VERTIGO

Neuroimaging is still considered the gold standard diagnostic test for stroke. However, computed tomography (CT) has very low sensitivity for stroke (approximately 16% in the first 24 h after stroke onset) so it is of little use for identifying acute ischemic strokes, particularly in the posterior fossa [19,20]. Despite this, nearly 50% of US emergency department patients presenting with dizziness undergo CT imaging [4]. Even diffusion-weighted MRI (DW-MRI), considered the gold standard for acute stroke diagnosis, misses up to 20% of acute posterior circulation strokes if performed less than 24 h from symptom onset [21]. Of the acute dizziness/vertigo cases in the emergency department, an estimated 40% of underlying strokes are missed. However, requesting neuroimaging to detect stroke amongst all patients with acute vertigo, most of whom will not have a stroke, presents a substantial challenge with high cost and resource implications [22]. Whilst noninvasive, bedside physical examination tests such as HINTS plus rarely miss stroke [14,23], this is only true for syndromes in which nystagmus is present, and when the assessment is carried out by an expert. Less attention has been given to the use of a focused neurological assessment to ‘screen out’ central neurological causes of acute vertigo that is of course inexpensive [24,25].

Neuroimaging with extracranial vessels is indicated in all patients with suspected posterior circulation stroke. However, even if the imaging is normal, ipsilateral hearing loss could point to a labyrinthine infarct and such patients should undergo further diagnostic testing and risk reduction management of future stroke – similar to patients with an acute unilateral visual loss secondary to retinal artery occlusion [26]. Evidence for thrombolysis in the absence of a thrombus is not known, and we would not currently advocate such interventions where the benefits are not known [27].

Posterior circulation TIA that presents with an isolated vestibular syndrome is a major diagnostic challenge [28]. We know that transient vestibular symptoms with positive diffusion-weighted lesions are associated with increased stroke risk within the week and months ahead [29] but it is not clear if such presentations without lesions of diffusion-weighted MRI carry any less risk. Approximately 15% of posterior circulation strokes are preceded by TIAs, and differentiating these from benign causes such as acute vestibular migraine remains a challenge [30,31^▪▪^].

As noted, early identification of vascular disorder such as thrombus or arterial dissection relies heavily on CT or MR angiography of the extracranial vessels [20]. In cases where a posterior circulation TIA is suspected, a clinical history indicating a sudden onset with peak symptom intensity occurring within seconds to minutes can aid in distinguishing it from vestibular neuritis, where the onset is typically over minutes. This distinction becomes particularly useful when patients present with isolated truncal ataxia in the absence of nystagmus, where vestibulr neutits is far less likely. When stroke or TIA remains high on the differential diagnosis list, it is prudent to act promptly: initiating antiplatelet agents, lipid-lowering therapy, and blood pressure control should be considered early, especially in individuals with known large-vessel atherosclerosis, given the elevated risk of recurrent cerebrovascular events.

## ACUTE VERTIGO PROTOCOLS

Hyperacute assessment (minutes or hours) of the dizzy patient represents one of the greatest clinical challenges in a patient with acute vertigo [32]. Provision of a protocol that recognizes that preservation of life and ‘brain’ is paramount above all and suggesting closer monitoring in the 24–48 h after initial presentation of symptoms would standardize care for patients with acute vertigo. Such guidance might recommend that a secondary prevention measure is instigated in patients with possible posterior circulation stroke, and appropriate investigations performed (e.g. vascular imaging for vertebral artery dissection) even if MRI-DWI imaging is normal.

Our real-world (unpublished) observations highlight the clinical implications of missed diagnostic opportunities. In a cohort of 71 patients presenting to the emergency department with vertigo or dizziness, only seven reported any auditory symptoms, and none underwent formal hearing evaluations at the time [33^▪^]. Subsequent pure-tone audiometry revealed asymmetrical sensorineural hearing loss in 11 individuals, six of whom were later diagnosed with acute vascular events that had initially been overlooked [33^▪^]. The prevalence of acute hearing loss in AICA infarction varies considerably in published literature – likely a reflection of how rarely audiometry is employed in acute settings and how often patients fail to perceive mild hearing deficits during episodes of intense vertigo and nausea. Portable audiometry, which is validated, low-cost, and easy to use with minimal training requirements, provides a promising avenue for improving detection.

Several diagnostic algorithms exist for acute vertigo, aiming to differentiate between peripheral and central causes. Key algorithms include HINTS, STANDING, and TiTrATE, often combined with other tests like the Dix–Hallpike manoeuvre for BPPV, bedside eye movement assessments, and standing balance. While these algorithms are of clinical value in the correct context, it is important to highlight that no single test is perfect, and whilst some may have higher sensitivity or specificity than others, they are best utilized in conjunction with a focused clinical history and neurological assessment.

A recent study evaluated the ‘TiTrATE - STANDING Adapted’ algorithm for diagnosing acute vertigo in emergency settings, aiming to assist nonspecialist clinicians in identifying posterior circulation strokes [34]. While the algorithm showed high sensitivity (90%), its low specificity (57.9%) led to frequent false positives, particularly in cases of vestibular migraine and chronic vascular conditions. The findings suggest that integrating TiTrATE, HINTS Plus and STANDING could improve diagnostic accuracy, but further refinement and validation – especially regarding vestibular migraine and stroke exclusion – are needed before clinical implementation [34].

## REMOTE ASSESSMENT

Remote assessment of acute vestibular symptoms is challenging, particularly when patients are severely unwell and evaluations must be conducted via telephone referral. Encourage clinicians to avoid using ambiguous terms like ‘HINTS positive’ or ‘negative’ and instead describe observed findings in detail. Video recordings of eye movements can be especially valuable, as signs in AVS can evolve rapidly, and video allows for repeated, slowed-down analysis. Simple screening questions – such as whether the patient can walk or sit upright or has acute unilateral hearing loss – can help flag serious disorder. While TeleStroke networks [35] have proven effective and cost-efficient in stroke care, applying telemedicine to acute vertigo remains limited in the UK due to a shortage of neuro-otology specialists, making widespread implementation feasible only in select tertiary centres.

## TECHNOLOGICAL ADVANCES FOR ACUTE VERTIGO

Eye recording technology to capture and analyse eye movements can aid the interpretation of any nystagmus in patients with acute vertigo [36]. Video recording (with expensive oculography or more accessible smartphone technology) can assist in the identification of subtleties of eye movements, as the recordings can be played back at slower speeds. Advancements in video goggles to include a gyroscope, and accelerometer measurements can also aid in the quality and standardization of assessments such as HINTS plus and the Dix–Hallpike manoeuvres. Further advancements may include the use of smartphones for eye movement recording and interpretation [37]. Expansions in algorithms and artificial intelligence in medical practice that could offer triaging or screening in this setting may offer enhanced diagnostic consistency and standardized care [38^▪▪^,^39^,^40^].

## CONCLUSION

The accurate evaluation of patients with acute vertigo requires a structured and focused clinical approach. A detailed history, combined with bedside assessment of eye movements, hearing, gait and truncal ataxia, can provide critical diagnostic clues to distinguish peripheral from central causes. While vascular imaging plays an important role, clinicians must be aware of its limitations, particularly in the early phase of posterior circulation stroke but also with labyrinthine ischaemia, where strokes may be too small for current imaging protocols to detect. The integration of validated bedside tests with emerging technologies – such as smartphone-based eye movement recording – offers a promising avenue to support real-time decision-making, improve diagnostic confidence, and ultimately reduce the risk of misdiagnosis and delayed treatment.

## Acknowledgements

*None*.

### Financial support and sponsorship

*None.*

### Conflicts of interest

*There are no conflicts of interest.*

## REFERENCES AND RECOMMENDED READING

Papers of particular interest, published within the annual period of review, have been highlighted as:

▪ of special interest
▪▪ of outstanding interest
